# Optimization of Extraction Conditions from Gac Fruit and Utilization of Peel-Derived Biochar for Crystal Violet Dye Removal

**DOI:** 10.3390/molecules29143435

**Published:** 2024-07-22

**Authors:** Nhat-Thien Nguyen, Pin-Ru Chen, Ru-Hau Ye, Kai-Jen Chuang, Chang-Tang Chang, Gui-Bing Hong

**Affiliations:** 1 Department of Chemical Engineering and Biotechnology, National Taipei University of Technology, No. 1, Sec. 3, Zhongxiao E. Rd., Taipei 106, Taiwan; nguyennhatthien333@gmail.com (N.-T.N.); slulu8673@gmail.com (P.-R.C.); k050614@gmail.com (R.-H.Y.); 2 School of Public Health, College of Public Health and Nutrition, Taipei Medical University, Taipei 110, Taiwan; kjc@tmu.edu.tw; 3 Department of Public Health, School of Medicine, College of Medicine, Taipei Medical University, Taipei 110, Taiwan; 4 Department of Environmental Engineering, National Ilan University, Yilan City 260, Taiwan

**Keywords:** extraction, *Momordica cochinchinensis* Spreng., adsorbent, crystal violet

## Abstract

Gac fruit (*Momordica cochinchinensis* Spreng.) is a prominent source of carotenoids, renowned for its exceptional concentration of these compounds. This study focuses on optimizing the extraction of active components from the aril of gac fruit by evaluating the effects of extraction temperature, solid–liquid ratio, and extraction time. The primary objective is to maximize the yield of gac oil while assessing its antioxidant capacity. To analyze the kinetics of the solid–liquid extraction process, both first-order and second-order kinetic models were employed, with the second-order model providing the best fit for the experimental data. In addition, the potential of gac fruit peel as a precursor for biochar production was investigated through carbonization. The resultant biochars were evaluated for their efficacy in adsorbing crystal violet (CV) dye from aqueous solutions. The adsorption efficiency of the biochars was found to be dependent on the carbonization temperature, with the highest efficiency observed for BCMC550 (91.72%), followed by BCM450 (81.35%), BCMC350 (78.35%), and BCMC250 (54.43%). The adsorption isotherm data conformed well to the Langmuir isotherm model, indicating monolayer adsorption behavior. Moreover, the adsorption kinetics were best described by the pseudo-second-order model. These findings underscore the potential of gac fruit and its byproducts for diverse industrial and environmental applications, highlighting the dual benefits of optimizing gac oil extraction and utilizing the peel for effective dye removal.

## 1. Introduction

*Momordica cochinchinensis* Spreng., commonly referred to as gac fruit, is a perennial plant of the genus Momordica within the Cucurbitaceae family. This botanical species was initially documented as *Muricia cochinchinensis* by the Portuguese missionary Loureiro during his exploration of Vietnam in 1790. Subsequently, Sprengel reassigned the species to the Linnaean genus Momordica, leading to the alteration of its scientific nomenclature in 1826 [1]. Indigenous to regions including Vietnam, Thailand, India, Southwest China, and Taiwan, gac fruit is distinguished by its exceptional lycopene content, exceeding that of tomatoes by over 70 times, and its abundance in carotenoids, vitamin E, and other essential constituents [2]. Afterwards, the cultivation of Southeast Asian varieties of gac fruit was introduced to Taiwan, where it was planted extensively in the central and southern regions. However, the native gac fruit from Taitung is renowned for its superior quality, characterized by its sweet and mildly warm flavor, absence of oiliness, and lack of bitterness, setting it apart from its Southeast Asian counterparts. Comprising the exocarp, pulp, aril, seed, and connective tissue, gac fruit stands out as the fruit with the highest carotenoid content among all natural fruits and vegetables known to date. The aril of the gac fruit, particularly rich in lycopene and β-carotene, is esteemed for its nutrient density, earning it the monikers “super fruit” or “heaven’s fruit” [3].

Carotenoids are typically categorized into two groups: carotenes, which lack oxygen atoms and primarily include α-carotene, β-carotene, γ-carotene, and lycopene; and oxygen-containing carotenoids, such as lutein, zeaxanthin, and others. Due to their numerous physiological functions, including anti-inflammatory, antioxidant, cancer-preventive, vision maintenance, immune modulation, neuroprotective, and more, carotenoids have garnered significant attention from both domestic and international researchers, with a particular focus on lycopene and β-carotene [4]. There are various techniques available for extracting active ingredients, including distillation, cold pressure, solvent extraction [5], ultrasonic-assisted extraction [6], microwave-assisted extraction [7], and supercritical fluid extraction [8]. Among these, solvent extraction stands out as the most widely employed method for extracting vegetable oils and active compounds due to its simplicity, stability of extracts, high efficiency, and cost-effectiveness. Gac fruit is known for its high nutritional value and bioactive compounds, such as carotenoids and antioxidants [7]. Various extraction methods have been employed to maximize the yield and preserve the quality of these compounds. For instance, Wong et al. [6] reported on the efficiency of ultrasonic-assisted extraction for enhancing the extraction of carotenoids from gac fruit, demonstrating its potential for various applications.

Biochar derived from agricultural waste has garnered significant interest due to its potential for environmental remediation. Research has shown that biochar from various biomass sources can effectively remove contaminants from water, including dyes, heavy metals, and organic pollutants [9]. Crystal violet (CV) is a synthetic dye widely used in industries such as textiles, paper, and printing. It is known for its high stability and resistance to biodegradation, making it a persistent environmental pollutant. Numerous studies have demonstrated the efficacy of biochar in adsorbing dye molecules from aqueous solutions. CV dye, in particular, is frequently used as a model pollutant to evaluate the adsorption capacity of biochar. For example, Tran et al. [10] investigated the adsorption of CV using biochar derived from tea waste and observed significant removal efficiency, attributing it to the surface area and functional groups of the biochar. The removal of CV from wastewater is therefore of significant environmental concern [11]. Numerous studies have utilized CV dye as a model contaminant to evaluate the adsorption efficiency of various adsorbents. This facilitates the comparison of the adsorption performance of biochar from gac fruit peel with other adsorbents reported in the literature [12,13].

The extraction of antioxidant components from plants typically follows a solid–liquid extraction process. The determination of extraction kinetic parameters is crucial for designing an efficient extraction process [14,15]. Various solid–liquid extraction kinetic models are commonly used, including the Ponomaryov empirical formula [16], Peleg model [17], Fick diffusion law [18], and first-order kinetic models [19,20]. Despite numerous studies on antioxidant components extracted from natural plants, there is a notable dearth of data assessing the extraction kinetic models used for this purpose [21]. Therefore, this study focused on extracting arils from native gac fruit in Taitung and repurposing the residual peel of gac fruit, which is often discarded as waste, to create a value-added product—biochar. Due to their cost-effectiveness, ample availability, and potential for recycling, there is a viable opportunity to convert agricultural waste and by-products into adsorbent materials with enhanced value. The solid–liquid extraction process and the performance of biochar in CV adsorption will be evaluated using mathematical models.

## 2. Results and Discussion

### 2.1. Extraction Results

#### 2.1.1. Effect of Extraction Temperature

**Figure 1:**
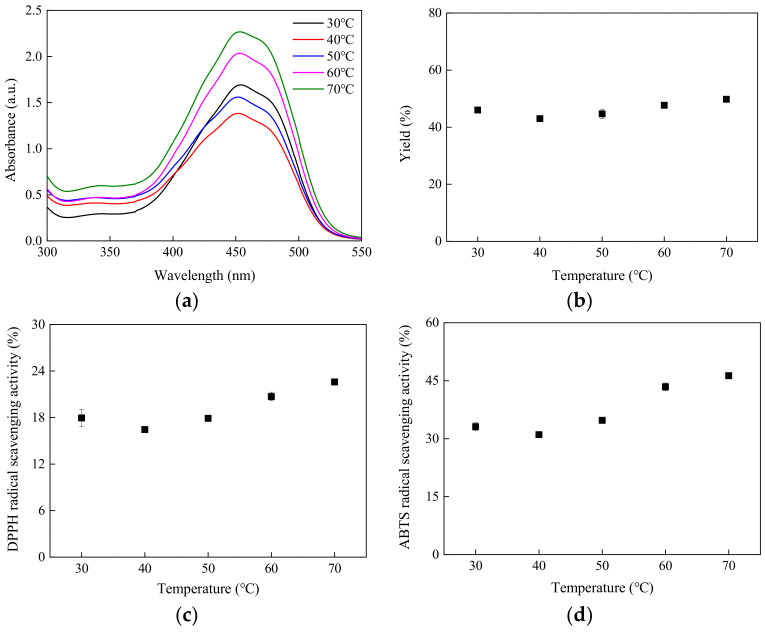
Temperature effect on (**a**) UV/vis spectrum, (**b**) extraction yield, (**c**) DPPH free radical scavenging ability, and (**d**) ABTS free radical scavenging ability.

In accordance with the findings of Lan et al. [22], lycopene and β-carotene exhibit notable absorption peaks in the UV/vis spectrum within the 400–500 nm range. These peaks are consistent with the characteristic absorption peaks of lycopene and, specifically, with those of β-carotene at 452 nm and 478 nm. As depicted in Figure 1a, at a solid–liquid ratio (S/L ratio) of 1/9 and an extraction duration of 30 min, the absorption intensity of the extract obtained at temperatures below 50 °C approached a similar level. However, the peak around 450 nm exhibited a significant increase as the temperature surpassed 50 °C, indicating an increase in lycopene content in the extract during the extraction process. As the temperature rose from 50 °C to 60 °C, carotenoid recovery increased rapidly, facilitated by the faster and easier removal of fatty acids from the cell wall and particles, leading to higher carotenoid concentrations. Correspondingly, the extraction yield, as well as the DPPH and ABTS radical scavenging abilities, exhibited an increase with the elevation of extraction temperature, as illustrated in Figure 1b–d. Elevating the extraction temperature can disrupt the phenolic-matrix bond and impact the membrane structure of plant cells, thereby enhancing the extraction efficiency of phenolic compounds. Nevertheless, excessively high extraction temperatures during heating may lead to the degradation of phenolic compounds, resulting in greater losses of components [23]. Consequently, subsequent extraction experiments were conducted at a constant temperature of 70 °C.

#### 2.1.2. Effect of S/L Ratio

**Figure 2:**
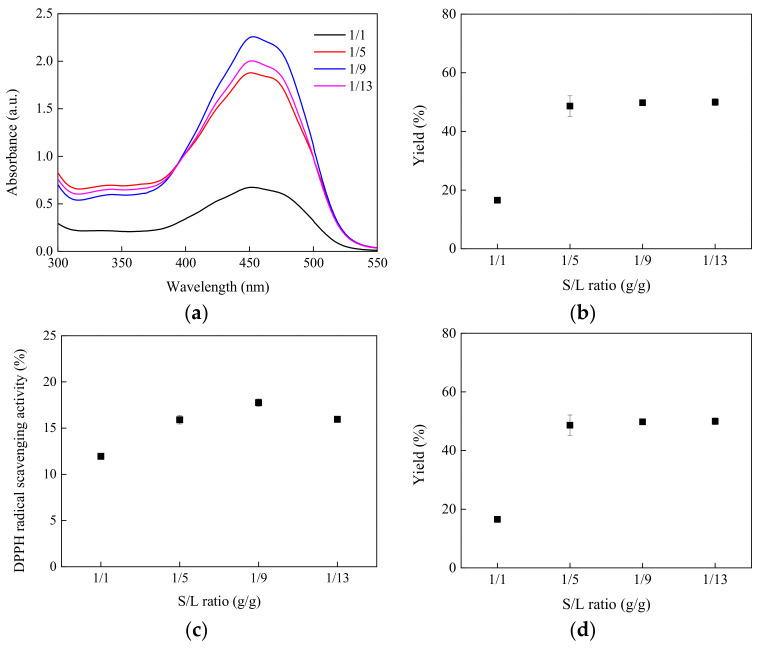
S/L ratio effect on (**a**) UV/vis spectrum, (**b**) extraction yield, (**c**) DPPH free radical scavenging ability, and (**d**) ABTS free radical scavenging ability.

The S/L ratio exerts a notable influence on the extraction process. The solvent plays a crucial role in expanding the material being extracted, necessitating complete immersion of the material in a sufficient volume of solvent to enhance extraction efficiency. Under the experimental conditions of a 70 °C extraction temperature and a 30 min extraction duration, the impact of different S/L ratios is illustrated in Figure 2. As depicted in Figure 2a, with the S/L ratio gradually increasing from 1/1 to 1/5 and 1/9, there is a significant rise in the absorbance intensity of the extracted material within the 400–500 nm range. However, the adsorption strength starts to decline upon further increase in solvent volume. Generally, a higher solvent volume increases the likelihood of penetrating plant cells, potentially leading to higher yields of active ingredients. Nonetheless, excessive solvent may reduce the extraction of active ingredients due to inadequate stirring [24]. Similarly, it is evident that the extraction yield, as well as the DPPH and ABTS free radical scavenging capacities, increase with rising solvent volume until an S/L ratio of 1/13, beyond which they begin to decline, as depicted in Figure 2b–d.

#### 2.1.3. Extraction Time Effect

**Figure 3:**
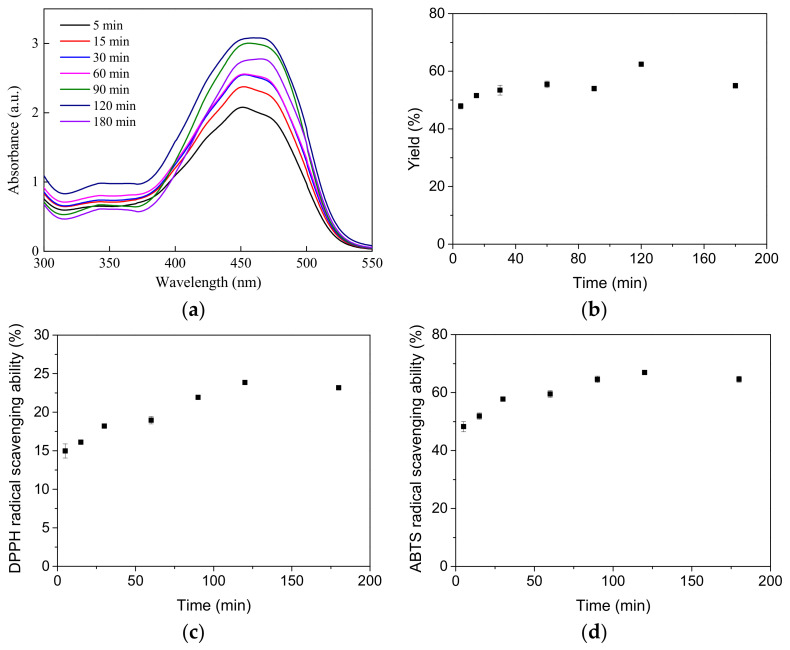
Extraction time effect on (**a**) UV/vis spectrum, (**b**) extraction yield, (**c**) DPPH free radical scavenging ability, (**d**) ABTS free radical scavenging ability.

The choice of extraction solvent polarity determines the substances that can be dissolved during the extraction process. In this experiment, ethanol, known for its lower toxicity, was utilized as the extraction solvent. Operating under conditions of 70 °C and an S/L ratio of 1/9, the impact of varying extraction times is illustrated in Figure 3. With an increase in extraction time, the absorption intensity (Figure 3a) of the extract in the UV/vis spectrum at 400–500 nm progressively rises, peaking at 120 min before declining. This trend is mirrored in the extraction yield, as well as the DPPH and ABTS radical scavenging capacities, as depicted in Figure 3b–d.

#### 2.1.4. Extraction Kinetics Data Calculation

**Figure 4:**
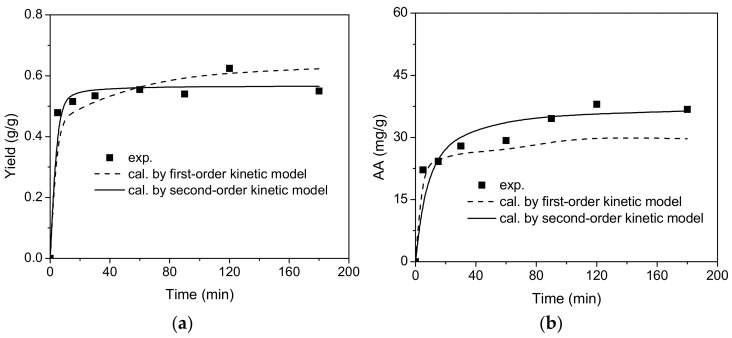
Extraction kinetics experimental results: (**a**) yield vs. time and (**b**) AA vs. time.

Extraction kinetics experiments were conducted to elucidate the relationship between extraction yield and ascorbic acid (AA) equivalent over time, under conditions of an S/L ratio of 1/9 and a temperature of 70 °C. The experimental findings are presented in Figure 4. Both first-order and second-order kinetic models were employed to depict the extraction kinetics data. The results indicated that the second-order kinetic model provided a better fit (R^2^ > 0.98) for both the extraction yield and ascorbic acid equivalent values compared to the first-order kinetic model. The parameters and model fitting results for each model are detailed in Table 1. Consequently, the extraction kinetics behavior of gac fruit can be suitably characterized using the second-order kinetic model.

### 2.2. Characterization of Biochars

#### 2.2.1. EA Result

To analyze the carbon proportions of gac fruit peel after carbonization at varying pyrolysis temperatures, an elemental analysis (EA) was utilized to determine the C, H, N, and S content of the biochars, as presented in Table 2. The carbon proportions of the biochars at different pyrolysis temperatures were 52.09%, 57.82%, 59.10%, and 62.98%, respectively, while the hydrogen (H) proportions were 4.769%, 2.898%, 2.030%, and 1.391%, respectively. The nitrogen (N) proportions were 1.95%, 1.86%, 1.66%, and 1.81%, respectively, and the sulfur (S) proportions were 0.00%, 1.324%, 0.637%, and 0.432%, respectively. The carbon content increased with rising pyrolysis temperature, reaching approximately 62.98% at 550 °C. Conversely, the proportions of H, N, and S decreased gradually with increasing pyrolysis temperature. The significant increase in C content, from 52.09% to 62.98%, suggests that most non-C elements in the gac fruit peel were volatilized during anaerobic roasting at pyrolysis temperatures ranging from 250 to 550 °C. The N proportion decreased by only 0.14%, from 1.95% to 1.81%, indicating that a substantial amount of N elements can be retained during low- to medium-temperature carbonization. Furthermore, the proportions of H decreased from 4.769% to 1.391%, while S decreased from 1.510% to 0.432%, showing limited variations.

#### 2.2.2. SEM/EDS Analysis Result

**Figure 5:**
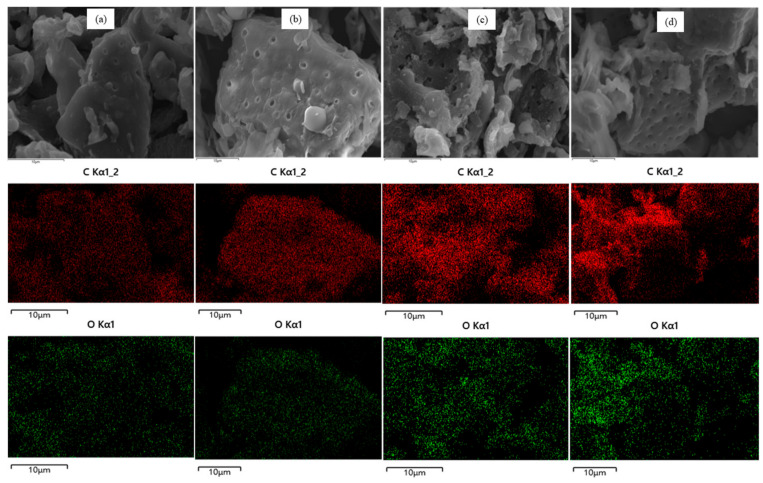
SEM/EDS analysis result of (**a**) BCMC250, (**b**) BCMC350, (**c**) BCMC450, and (**d**) BCMC550.

To elucidate the surface morphology and elemental composition of carbonized gac fruit peel, an SEM/EDS analysis (Figure 5) was conducted. The EDS mapping results indicate a strong dominance of carbon on the biochar surface during pyrolysis. The combined SEM and EDS results reveal that pyrolysis produces a rougher oxygen surface with varied pores, which is favorable for the adsorption of CV dye. At a carbonization temperature of 250 °C, the biochar began to develop pores, although their number was not significant. As the temperature increased to 350 °C and beyond, the carbonization process exhibited improved efficacy, resulting in a greater number of evenly distributed pores. These findings indicate that the optimal carbonization condition was achieved at 550 °C.

#### 2.2.3. FTIR Analysis Results

**Figure 6:**
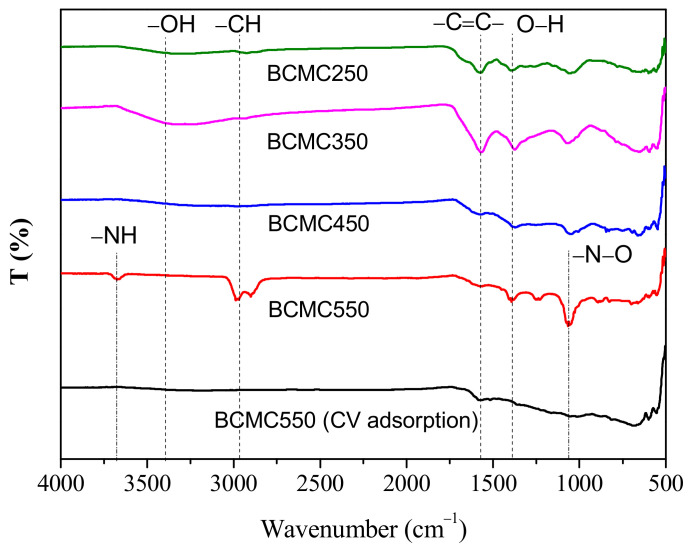
FTIR analysis of biochars.

Fourier-transform infrared spectroscopy (FTIR) was employed to analyze the functional groups present in the biochar derived from gac fruit peel. Figure 6 illustrates the variations in functional groups at different pyrolysis temperatures. The characteristic peaks of the CH_2_ functional group, corresponding to the bending vibration of the C–H bond, were identified at 2930 cm^−1^ in the biochar [25]. The other peak at 1378 cm^−1^ shows the O–H bonding of phenolic functional group [26]. The peak intensity associated with the vibration of the phenolic group at approximately 3500 cm^−1^ exhibits a slight, albeit not significant, decrease with an increase in pyrolysis temperature. Furthermore, with increasing pyrolysis temperature, the intensity of certain functional groups diminishes or even disappears [27]. For instance, the bending vibration corresponding to the C = C bond near 1580 cm^−1^ [28] conspicuously decreases as the pyrolysis temperature rises. Upon comparing the FTIR spectra of BCMC550 before and after CV adsorption, the disappearance of bands at 3750 cm^−1^ (–NH), 2930 cm¹ (C–H), and 1010 cm^−1^ (N–O) confirms that CV effectively interacted with the functional groups on the biochar surface [27].

#### 2.2.4. BET Analysis Results

To investigate the surface area, pore size, and pore volume of biochars derived from gac fruit peel at various carbonization temperatures, nitrogen adsorption–desorption isotherms were employed. The specific surface area, pore size, and pore volume of the biochars were determined, and the results are summarized in Table 3. The specific surface areas of BCMC250, BCMC350, BCMC450, and BCMC550 were found to be 296, 313, 528, and 697 m^2^·g^−1^, respectively. Correspondingly, the pore volumes were measured at 0.13, 0.18, 0.27, and 0.31 cm^3^·g^−1^, respectively, with pore sizes of 2.05, 2.03, 2.07, and 2.03 nm, respectively. The biochars exhibited the highest specific surface area and pore volume for BCMC550, followed by BCMC450, with the smallest observed for BCMC250. This suggests that gac fruit peel undergoes optimal carbonization near 550 °C, as temperatures exceeding this may compromise the surface structure of the biochar, while temperatures lower than 450 °C may not produce a sufficient number of pores.

**Figure 7:**
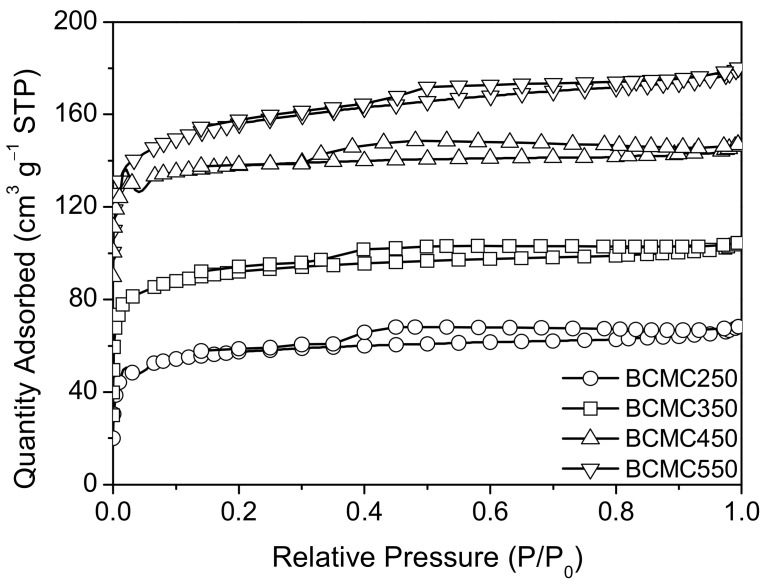
Nitrogen adsorption–desorption isotherms of biochars.

Figure 7 displays the nitrogen adsorption–desorption isotherms of biochar at different carbonization temperatures, illustrating that the N_2_ adsorption/desorption isotherms of BCMC250, BCMC350, BCMC450, and BCMC550 conform to Type IV isotherms for mesoporous materials [29]. Overall, the biochar produced at 550 °C was found to be optimal, exhibiting a specific surface area of 697 m^2^·g^−1^ and a pore volume of 0.31 cm^3^·g^−1^.

### 2.3. CV Adsorption Results

#### 2.3.1. CV Dye Adsorption onto Different Biochars

**Figure 8:**
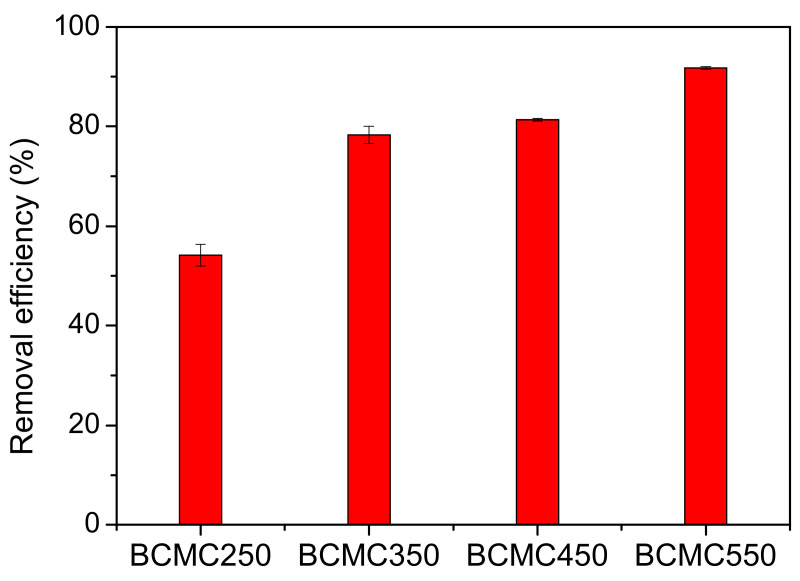
Adsorption results of CV dye onto biochars (*p* < 0.05).

To assess the adsorption capacity of biochar at different carbonization temperatures for CV dye, the adsorption performance of BCMC250, BCMC350, BCM450, and BCMC550 was compared. The adsorption experiments were conducted at 30 °C, with a CV dye concentration of 200 ppm, an adsorbent dose of 0.5 g·L^−1^, an adsorption time of 135 min, and a stirring speed of 100 rpm. A Shapiro–Wilk test was used to determine for the normality of continuous variable. An analysis of variance (ANOVA) was utilized for between-group comparisons among different biochars, with a significance level of 0.05 and a two-sided distribution determining the statistical significance in our models. The results revealed that the CV removal efficiencies of BCMC250, BCMC350, BCM450, and BCMC550 were 54.43%, 78.35%, 81.35%, and 91.72%, respectively, as illustrated in Figure 8 (*p* value < 0.05). The error bars in Figure 8 represent standard deviations. Among the biochars, BCMC550 exhibited the highest CV removal efficiency, attributed to the presence of functional groups on its surface that enhance CV dye adsorption, as indicated by the FTIR results (Figure 6). Furthermore, BCMC550 possessed the largest specific surface area (697 m^2^·g^−1^), the highest pore volume (0.31 cm^3^·g^−1^), and the highest carbon content (62.98%), all of which contribute to its superior adsorption performance.

#### 2.3.2. Adsorption Kinetics Data of CV Dye Adsorption onto BCMC550

**Figure 9:**
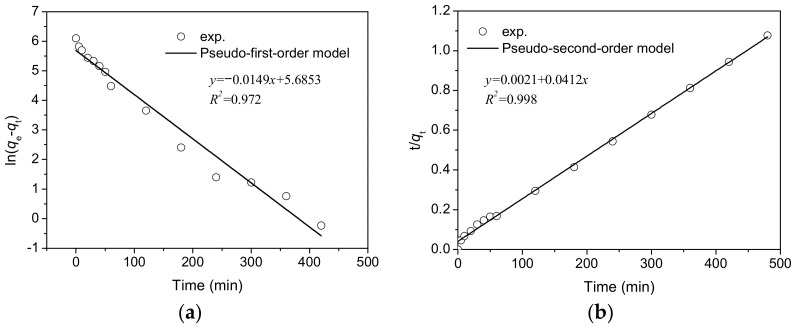
Correlation results of adsorption kinetics models: (**a**) pseudo-first-order; (**b**) pseudo-second-order.

To investigate the adsorption capacity of BCMC550 biochar for CV dye following carbonization at 550 °C, adsorption kinetic experiments were conducted under the following conditions: an adsorption temperature of 30 °C, a CV concentration of 200 ppm, a BCMC550 dosage of 0.5 g·L^−1^, an adsorption time ranging from 0 to 480 min, and a stirring speed of 100 rpm. The adsorption kinetics data were analyzed by the pseudo-first-order [30] and pseudo-second-order [31] kinetic models to assess the most appropriate kinetic model. The correlated results, depicted in Figure 9a, b, revealed that the pseudo-second-order model exhibited a superior fit, with a correlation coefficient (R^2^) value of 0.998, compared to the pseudo-first-order model, indicating its suitability for describing the adsorption of CV onto BCMC550. Table 4 provides a summary of the adsorption kinetics parameters derived from the adsorption kinetic models.

#### 2.3.3. Adsorption Isotherm Data of CV Dye Adsorption onto BCMC550

The adsorption isotherm data of CV adsorption onto BCMC550 were analyzed using the Langmuir [32] and Freundlich [33] adsorption isotherm models, as detailed in Table 5. The correlation results indicated that the Langmuir isotherm model exhibited a higher linear regression coefficient (R^2^) compared to the Freundlich model, suggesting that the Langmuir model is more suitable for describing the isothermal adsorption behavior of CV onto BCMC550. The Langmuir adsorption isotherm model assumes monolayer coverage, negligible interactions between adsorbate molecules on adjacent sites, and uniform adsorption energy, thereby illustrating the adsorption behavior. This result suggests that CV dye is adsorbed onto the uniform surface of BCMC550, forming a monolayer with uniform adsorption energy. According to the Langmuir model, the maximum monolayer adsorption capacity (*q_m_*) of BCMC550 biochar for CV is calculated to be 909.1 mg·g^−1^. Furthermore, the adsorption capacity of BCMC550 was observed to increase with an increasing temperature.

Table 6 provides a comparison of CV adsorption capacities on different biosorbents with those reported in previous studies. It can be observed that BCMC550 exhibits a significantly higher adsorption capacity compared to other adsorbents, classifying it as one of the most effective adsorbents in this context. The high maximum adsorption capacity (*q*_max_ = 909.1 mg·g⁻¹) can be attributed to the cationic nature of CV, which readily adsorbs onto the functional groups present on the biochar surface. Furthermore, morphological studies revealed that BCMC550, obtained through thermal flash pyrolysis, is a well-developed porous material that allows CV dye to diffuse into the internal pore network of the biochar and become immobilized. The enhanced adsorption efficiency observed in this system is attributed to the pyrolysis process, which produces a rougher oxygen-rich surface with varied pore sizes, favoring the adsorption of CV dye.

## 3. Materials and Methods

### 3.1. Materials

Gac fruit was sourced from local farms in Taitung County, Taiwan. Methanol (≥99.5%) and ethanol (≥99.8%) were obtained from Fisher Chemical, Hampton, NH, USA. Ascorbic acid (≥99%), crystal violet (CV) and potassium persulfate (≥99%) were supplied by Acros Organics, Geel, Belgium. DPPH (2,2-diphenyl-1-picrylhydrazyl) (≥95%) was purchased from Alfa Aesar, Ward Hill, MA, USA, while ABTS (2,2’-azino-bis (3-ethylbenzothiazoline-6-sulphonic acid)) (≧98%) was procured from Sigma-Aldrich, St. Louis, MO, USA. All chemicals were of HPLC grade and utilized without further purification.

### 3.2. Extraction Method

#### 3.2.1. Pre-Treatment of Gac Fruit

**Figure 10:**
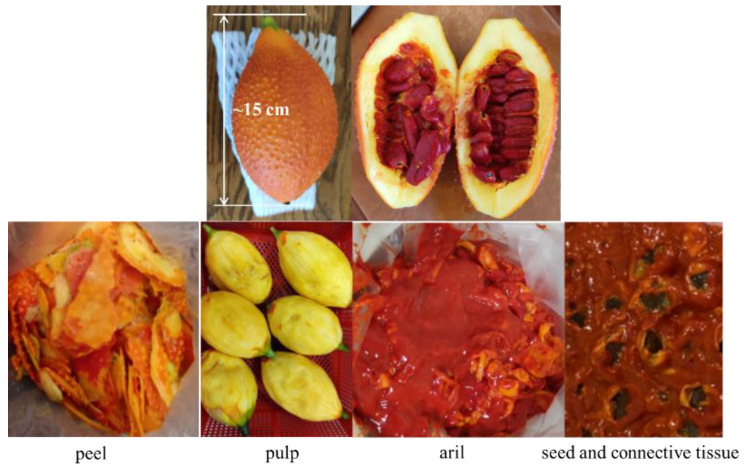
Anatomy of gac fruit.

Slice open the entire native gac fruit and segregate the pulp, peel, aril, and seeds, as shown in Figure 10. Subsequently, place the arils in a −20 °C refrigerator for a minimum of 3 days. Transfer the arils to a manifold freeze dryer set to a condensation temperature of −60 °C and vacuum pressure below 0.04 mbar for at least 7 days. Grind the dried arils into a powder using a mortar and sieve, aiming for a powder size of less than 20 mesh. Store the powdered arils in a sealed glass container and refrigerate at 4 °C to complete the pretreatment. Following pretreatment, the proportions of pulp, peel, aril, and seed of the total weight of gac fruit were measured at 55.7%, 13.0%, 17.4%, and 13.8%, respectively. The proportion of the aril was 17.4%, which falls within the reported range of 6% to 31% [41]. These variations may be attributed to differences in variety, growing conditions, and the maturity of the fruits investigated [42]. The aril exhibited an average dehydration rate of approximately 75.2%, indicating a high water content as characteristic of gac fruit.

#### 3.2.2. Extraction of Active Components

The aril powder was introduced into the extraction solvent, and the beaker was sealed with a film before being placed in a constant temperature shaking water bath set to a shaking frequency of 100 rpm. Following extraction, the resulting extract was filtered using Whatman No. 6 filter paper via vacuum filtration, and subsequently purified using a vacuum concentration device with condensation set at 5 °C and a water bath at 40 °C. The extract yield was calculated as (weight of extract/weight of dried material) × 100%. Various operational factors can influence the efficiency of solvent extraction, including pre-treatment methods, solvent types, solid-to-liquid ratio (S/L ratio), and extraction temperature. This study investigates the influence of different variables on the extraction efficiency and assesses the antioxidant capacity of the extract through DPPH and ABTS free radical scavenging assays.

#### 3.2.3. DPPH Free Radical Scavenging Assay

Following the method described by Shimada et al. [43] with some modifications, 50 mL of diluted extract was combined with 950 mL of 0.6 mM DPPH methanol solution and allowed to incubate at room temperature in darkness for 30 min. The absorbance at a wavelength of 515 nm was then measured using a UV/vis spectrophotometer (Thermo Scientific, Braunschweig, Germany). The scavenging rate was calculated using the formula [1– (absorbance of sample at 515 nm/absorbance of blank at 515 nm)] × 100%, and expressed as milligrams of ascorbic acid (AA) equivalents per gram of dry weight (mg AA equivalents·g^−1^). A standard curve was constructed using various concentrations of ascorbic acid as the standard.

#### 3.2.4. ABTS Free Radical Scavenging Assay

Adhering to the procedure outlined by Re et al. [44] with certain modifications, a 7.4 mM solution of ABTS and a 2.45 mM solution of potassium persulfate were prepared and combined in a 1:1 volume ratio and then left to incubate in darkness at room temperature for 12 h. Subsequently, the mixture was diluted with deionized water to achieve an absorbance of 1.0 ± 0.02. Then, 50 mL of the diluted extract was mixed with 950 mL of the ABTS solution and allowed to stand in darkness at room temperature for 30 min. The absorbance of the resulting mixture was measured at a wavelength of 750 nm using a UV/vis spectrophotometer. The scavenging rate was calculated as [1 − (absorbance of sample at 750 nm/absorbance of blank at 750 nm)] × 100%, and expressed as milligrams of ascorbic acid equivalents per gram of dry weight. Standard curves were constructed using various concentrations of ascorbic acid.

#### 3.2.5. Extraction Kinetics Models

The data derived from the extraction experiments were utilized to ascertain the extraction rate constants. The solid–liquid extraction kinetics models employed encompassed first-order kinetic models and second-order kinetic models. The first-order kinetic model [45] is expressed as follows:

(1) $$\frac{{dC}_{t}}{dt}=k_{1}(C_{e}-t)$$

After integrating and rearranging, we can obtain

(2) $$ln\left(\frac{C_{e}}{C_{e}-C_{t}}\right)=k_{1}t+a$$

where *k_1_* is the overall mass transfer coefficient; *a* is a semi-empirical constant related to the loss of active ingredients, plant structure, and absorbed water; *C_e_* is the equilibrium extraction yield (or ascorbic acid equivalent); and *C_t_* is the extraction yield (or ascorbic acid equivalent) at any time (*t*), with *k_1_* and *a* as the parameters to be determined.

The second-order kinetic model [14] posits that under steady-state conditions, the extraction concentration remains uniform, and the concentration of the extracted product remains consistent under identical extraction conditions. The extraction yield (or ascorbic acid equivalent) can be represented by the following equation:

(3) $$\frac{dC_{t}}{dt}=k_{2}{\left(C_{e}-C_{t}\right)}^{2}$$

After integration of Equation (3) under the conditions of *C_t_* = 0 at *t* = 0 and *C_t_* = *C_t_* at *t* = *t*, Equation (4) can be obtained:

(4) $$C_{t}=\frac{C_{e}^{2}k_{2}t}{1+C_{e}k_{2}t}$$

where *k_2_* is the second-order rate constant, and the parameters to be determined are *k_2_* and *C_e_*.

### 3.3. Preparation of Adsorbent

The gac fruit peel was converted into biochar carbon using a slow pyrolysis method. The specific procedure involved drying the collected peel using freeze-drying, followed by storage in sealed bags with labels. A certain quantity of the dried peel was then placed in a quartz tube (26 × 30 × 700 mm) and pyrolyzed under a nitrogen gas atmosphere. The tube furnace parameters were set with a heating rate of 5 °C·min^−1^, pyrolysis temperatures of 250, 350, 450, and 550 °C, and a pyrolysis time of 1 h. After cooling to room temperature, the carbonized samples were removed and stored in a dryer for future use. The resulting biochars derived from gac fruit peel after post-carbonization were labeled as BCMC250, BCMC350, BCMC450, and BCMC550, corresponding to the respective pyrolysis temperatures. Scanning electron microscopy/Energy-dispersive X-ray spectrometry (SEM/EDS), elemental analysis (EA), specific surface area analysis using the Brunauer–Emmett–Teller (BET) method, and Fourier transform infrared spectroscopy (FTIR) are employed for the characterization of biochars.

### 3.4. Dye Adsorption Experiment

In this investigation, CV dye served as the model pollutant. The adsorbent, at a dosage of 0.5 g·L^−1^, was immersed in a dye solution with an initial concentration of 200 ppm, and the adsorption process was conducted at a temperature of 30 °C. The system was then subjected to a constant temperature shaking water bath, and the adsorption kinetics of CV were monitored at intervals ranging from 0 to 480 min. The absorbance of CV dye on biochars was quantified using a UV/vis spectrophotometer at a peak wavelength of 590 nm, consistent with the approach of Sohni et al. [46]. The removal efficiency and adsorption capacity of the dye were computed according to the methodology outlined by Ma et al. [47]. The adsorption kinetic behavior of CV on the adsorbent was characterized using various adsorption kinetic models. Furthermore, the adsorbent, also at a dosage of 0.5 g·L^−1^, was exposed to varying concentrations of CV solutions, ranging from 200 to 600 ppm. These mixtures were similarly placed in a constant temperature shaking water bath, with the adsorption temperature set between 30 and 60 °C. The optimization of adsorbent dosage and adsorption temperature conditions aligns with the work of Kosale et al. [34]. The adsorption process was allowed to proceed for 180 min, after which the adsorption isotherm data of CV were collected and analyzed using adsorption isotherm models. All the experiments were repeated independently at least three times.

## 4. Conclusions

This study investigates the extraction of Taiwan’s native species of gac fruit, with a gac oil yield exceeding 50% and the highest antioxidant ability observed under the extraction conditions of 70 °C, a solid-to-liquid (S/L) ratio of 1/9, and an extraction time of 120 min. The peel of the gac fruit, an underutilized agricultural waste, was utilized to prepare biochar. This approach not only offers an eco-friendly solution to waste management but also explores a novel source material for biochar production and dye adsorption, which has not been extensively studied. The preparation of biochar from gac fruit peel involves environmentally benign processes, aligning with sustainable practices. The application of the waste for biochar production in dye removal studies indicates that BCMC550 biochar demonstrates the highest CV dye removal efficiency, attributed to its largest specific surface area (697 m^2^·g^−1^), highest total pore volume (0.31 cm^3^·g^−1^), and highest carbon content (62.98%). Our results demonstrated a significant adsorption capacity for CV dye, highlighting the efficacy of gac fruit peel biochar as an adsorbent. This suggests that BCMC550 may be effectively utilized to remove substantial amounts of cationic dyes from wastewater, in contrast to other adsorbents. This underscores the potential of converting waste materials into valuable adsorbents.

## Figures and Tables

**Table 1:** Calculation results of extraction kinetics models.

Models	Parameters	Yield	AA Equivalent
First-order kinetic model	*k*_1_ (min^−1^)	0.019	0.038
	*a*	1.144	0.120
	*R^2^*	0.406	0.813
Second-order kinetic model	*k*_2_ (g·g^−1^min^−1^)	2.610	0.004
	*C_e_*	0.568	37.736
	R^2^	0.994	0.981

**Table 2:** Elemental analysis results of biochars.

Biochars	N (%)	C (%)	H (%)	S (%)	C/N	C/H
BCMC250	1.95	52.09	4.769	1.510	26.73	10.92
BCMC350	1.86	57.82	2.898	1.324	31.05	19.95
BCMC450	1.66	59.10	2.030	0.637	35.63	29.12
BCMC550	1.81	62.98	1.391	0.432	34.77	45.28

**Table 3:** BET analysis results of biochars.

Biochars	Surface Area (m^2^·g^−1^)	Pore Volume (cm^3^·g^−1^)	Pore Size (nm)
BCMC250	295.79	0.13	2.05
BCMC350	312.89	0.18	2.03
BCMC450	528.16	0.27	2.07
BCMC550	696.91	0.31	2.03

**Table 4:** Adsorption kinetic parameters for CV adsorption onto BCMC550.

Model	Parameters	
Pseudo-first-order	*k*_1_ (min^−1^)	0.0149
	*q_e_* (mg·g^−1^)	294.51
	R^2^	0.974
Pseudo-second-order	*k*_2_ (g·mg^−1^·min^−1^)	0.0001
	*q_e_* (mg·g^−1^)	476.19
	R^2^	0.998

**Table 5:** Adsorption isotherm model parameters for CV adsorption onto BCMC550.

Temperature (°C)	30	40	50	60
Langmuir				
*q_m_* (mg·g^−1^)	625.0	714.3	714.3	909.1
*K_L_* (L·g^−1^)	0.123	0.187	0.636	0.239
*R_L_*	0.039	0.026	0.008	0.021
R^2^	0.997	0.994	0.997	0.998
Freundlich				
*K_F_* (mg·g^−1^)(L·mg^−1^)^1/zn^	276.4	376.8	384.2	724.4
*n*	15.55	7.800	7.525	6.821
R^2^	0.942	0.921	0.919	0.986

**Table 6:** Comparison of the CV adsorption capacities of different biosorbents.

Adsorbent	*q*_max_ (mg·g^−1^)	Reference
Activated carbon MO_CA-H3PO4_	469.55	[27]
Black Plum seed biochar (BPSB)	42.39	[34]
Fe_3_O_4_-coated biochar	349.4	[35]
Biochar (CB-LDH)	374.686	[36]
Woody tree biochar	125.5	[37]
Biochar derived from palm stalk	209	[38]
Fe_3_O_4_-graphene-rice straw-derived biochar	436.68	[39]
Magnetic biochar derived from rice straw	111.48	[40]
BCMC550	909.1	This study

## Data Availability

Data are contained within the article.
